# Accuracy of Three Impression Materials on the Totally Edentulous Maxilla: In Vitro/In Silico Comparative Analysis

**DOI:** 10.3390/ma13030515

**Published:** 2020-01-22

**Authors:** Fernando Zarone, Gennaro Ruggiero, Maria Irene Di Mauro, Gianrico Spagnuolo, Marco Ferrari, Roberto Sorrentino

**Affiliations:** 1 Department of Neurosciences, Reproductive and Odontostomatological Sciences, University “Federico II” of Naples, 80131 Naples, Italy; fernandozarone@mac.com (F.Z.); mariadimauro94@gmail.com (M.I.D.M.); gianrico.spagnuolo@gmail.com (G.S.); errestino@libero.it (R.S.); 2 Department of Medical Biotechnologies, Division of Restorative Dentistry and Endodontics, University of Siena, 53100 Siena, Italy; ferrarm@gmail.com

**Keywords:** polyether, polysulfide, polyvinyl siloxane, elastomer, edentulous maxilla impression, optical impression, digital dentistry, laboratory scanner, accuracy, digital model

## Abstract

Background: This study was aimed at comparing the accuracy of impressions of a reference typodont (RT) reproducing a totally edentulous maxilla made with three impression materials: polysulfide, polyether, and polyvinyl-siloxane. Methods: The RT was scanned using a desktop scanner, obtaining a reference scan. Ten impressions for each of the three tested materials were made using a mechanical device with a standardized and consistent modality. A laboratory scanner performed the digitization of each impression. We produced digital models by processing “in reverse” the scans of the physical impressions using a dedicated software, obtaining three groups (n = 10), respectively. The groups were titled: “polysulfide,” “polyvinyl-siloxane,” and “polyether”. The scans in .stl format were imported into Geomagic Control X and then compared to RT to evaluate the accuracy of each scan by calculating trueness and precision in µm. Recorded data were subjected to descriptive statistics. Results: Trueness (arithmetic proximity) values (95%CI) were: polysulfide = 249.9 (121.3–378.5), polyvinyl-siloxane = 216.8 (123.1–310.6), polyether = 291.1 (219.9–362.3). Precision values (95% CI) were: polysulfide = 261.9 (108.8–415), polyvinyl-siloxane = 209.4 (111.9–306.8), polyether = 283 (227.9–338.1). Statistically significant differences were not detected between the means of the experimental groups, both for trueness and precision. Conclusions: The accuracy of the scans obtained from polyvinyl-siloxane, polysulfide, and polyether impressions can be considered comparable in a fully edentulous maxilla.

## 1. Introduction

Edentulism, defined as “the absence or complete loss of all-natural dentition (teeth)” [1], is a debilitating and irreversible condition [2], considered as the “final marker of disease burden for oral health” [3].

To date, the oldest and most widespread treatment of total edentulism is the conventional complete denture [4,5,6], in which the first operative step is represented by a correct impression procedure [7]. The procedure can be accomplished using different techniques: mucostatic [8], mucocompressive, selective pressure [9], functional [10], and neutral zone impression [11]. Different impression materials have been suggested over time, such as polysulfide, polyvinyl siloxane, irreversible hydrocolloids, zinc-oxide eugenol pastes, and polyethers [7,12,13].

In the last years, there has been growing interest in a full-digital concept of complete dentures focusing on the use of optical impressions in the field of removable prosthodontics [14,15,16,17,18]. The protocol for a digitally produced complete denture starts with the digitization of an edentulous arch that can be performed using intraoral or laboratory scanners. The use of an intraoral scanner (IOS) does not require any physical gypsum model or physical impression. Instead, using a laboratory scanner, it is possible to obtain digitization by scanning the physical gypsum model obtained with a conventional impression procedure or by scanning the physical impression itself. In this last case, the file is reversed to obtain a positive reproduction of the digital model [19].

The present study was aimed at comparing the accuracy of scans obtained by digitizing impressions made with three different impression materials: polysulfide, polyether, and polyvinyl siloxane, on a reference typodont of a totally edentulous maxilla. 

The null hypothesis stated that there was no difference between the accuracy obtained by scanning each of the three different impression materials and that of the reference scan.

## 2. Materials and Methods 

### 2.1. Preparation of the Reference Typodont

**Figure 1:**
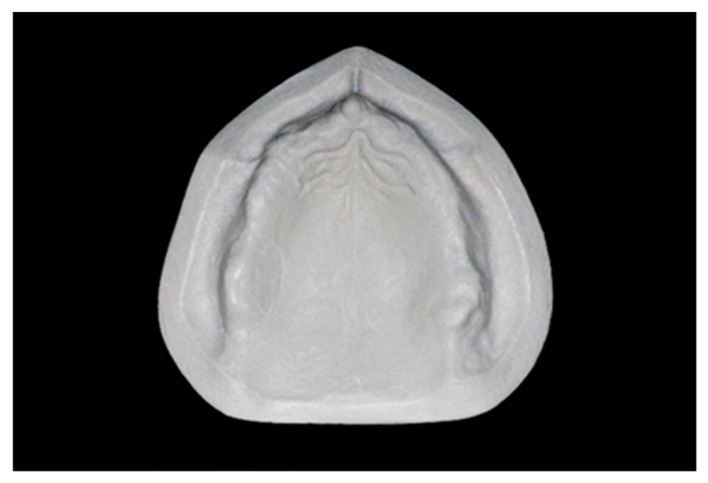
Reference typodont (RT) in polyurethane resin.

A real model of a totally edentulous maxilla, previously obtained from a dental patient, was duplicated using a dedicated silicone material (Elite Double; Zhermack SpA). We created a mold inside which polyurethane resin (PRIMA-DIE; Gerhò SPA) was poured with the purpose of fabricating the reference typodont (RT) (Figure 1).

A digital reference typodont (dRT) file was obtained and saved in .stl format, scanning the RT with a metrological desktop scanner (Atos Core 80; GOM, Braunschweig, Germany), based on a structured white-light technology, with the following settings: measure accuracy = ± 0.0025 mm, point spacing = 0.03 mm, and working distance = 170 mm.

### 2.2. Conventional Impression Procedure

**Figure 2:**
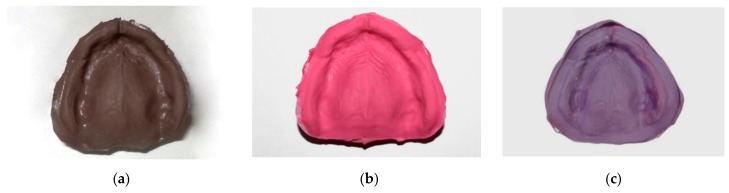
Physical impressions made of: (**a**) polysulfide; (**b**) polyvinyl siloxane; (**c**) polyether.

Ten impressions were made using each of the three different impression materials (Figure 2): polysulfide (Permlastic Regular body; Kerr, Orange, California, CA, USA), polyether (Impregum Penta medium-bodied; 3M ESPE, Maplewood, Minnesota, MN, USA), and polyvinyl siloxane (Vestige medium; Trayart srl, Padova, Italy), in a standardized and reproducible way. All the impressions were made by the same experienced prosthodontist, during the same morning and in the same room, under similar environmental conditions: temperature of 22 °C, air pressure of 760 ± 5 mmHg, and 45% relative humidity. Five initial impressions were made for each of the three impression materials and then discarded, accomplishing a training session.

To get predictable and consistent impression procedures, a custom impression tray was made first by placing a 3.0 mm layer of wax (Tenasyle; Kemdent, Swindon, England) onto the RT, as a spacer between the pre-formed light-curing resin base (ValSax; Capuozzo S.r.l., Naples, Italy) and the RT [20]. The margins of the impression tray were 2 mm short for the bottom of the buccal fornices, according to construction techniques of the custom tray employed for the impression procedure, described in the Passamonti’s protocol for the realization of a complete denture [21]. No handle was made on the impression tray, in that the tray was designed to be secured to a specific tester base using 3 cylinders protruding from the external surface of the tray itself, as reference positioning points. No tissue stops were made because the evenness of impression material thickness was guaranteed by the design and modality of the working of the tester. A duplication silicone (ADDISIL A + B 85; Bartolini Dental Group S.r.l., Terni, Italy) was used to create a mold of the reference tray, inside which a self-curing resin (BI CRYL COLD N A + B; Bartolini Dental Group S.r.l.) was cast to allow the duplication of the reference tray. The silicone and the resin were then placed in an electronic polymerizer with water at 55 °C and electronically controlled pressure at 5 × 105 Pa for 8 min. Using this method, 30 identical impression trays were fabricated and used after 48 h.

**Figure 3:**
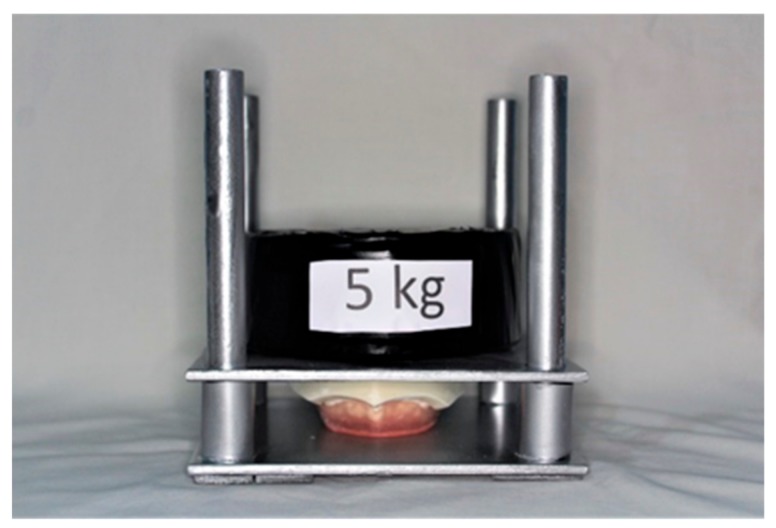
Standardized testing device for impression making.

The tester used in this study was a custom-made mechanical precision instrument, made of iron to guarantee precision and consistency of the impression procedures. It had a square support base (side = 17 cm), with 3 holes allowing the fitting of the 3 reference cylinders of the tray, keeping it fixed in a constant and stable position. The metal base supported 4 perpendicular cylinders of 16.5 cm in length and 1.5 cm in diameter, parallel to each other, lubricated with petroleum jelly, thereby allowing an upper, identical metal square plate to slide smoothly onto the base. The upper base had 3 holes through which the typodont could be blocked to its lower surface, on account of 3 passing screws. Four polyvinyl chloride (PVC) tubes of 3.1 cm in length, positioned around the iron cylinders, provided a mechanical stop to leave a 3.0 mm free space between the RT and the tray. A constant, repeatable pressure during impression making of the RT was guaranteed by a weight of 5 kg placed on the upper plate of the tester, aimed at pressing the typodont onto the impression tray containing the impression material (Figure 3).

In this study, the three impression materials were mixed following the instructions provided by the manufacturers. However, it is worth noting that the setting time in the present in vitro test was increased compared to the intra-oral environment, being the impressions made under different temperatures (22 °C) compared to the intra-oral conditions (35–36 °C) [22]. The following timetable was applied for each impression material:

- Polysulfide: manual mixing time = 50 s (mixing ratio 1:1); material placement into the tray and impression making = 20 s; removal of the impression tray from the tester = 15 min from the beginning of mixing;
- Polyvinyl siloxane: for initial use, a small amount of material was extruded for 5 s and then discarded; the extruded material was deleted, then it was mounted on the mixing tip; auto-mixing (mixing ratio 1:1); material placement into the tray and impression making = 30 s; removal of the impression tray from the tester = 15 min from the beginning of mixing;
- Polyether: for initial use, a small amount of material was extruded for 5 s and then discarded; auto-mixing (mixing ratio 5:1, 300 ml of base paste and 60 ml of catalyst paste); material placement into the tray and impression making = 30 s; removal of the impression tray from the tester = 15 min from the beginning of mixing.

All the elastomers were mixed by following the manufacturers’ instructions. The polysulfide was mixed manually using a stainless steel spatula on a glass slab; the polyvinyl siloxane was auto-mixed in a dedicated dispenser with two separate equally sized cylinders for the base paste and the catalyst paste and a mixing tip (Universal manual dispenser; Trayart srl); the polyether was auto-mixed in a motorized mixing device, with two parallel pistons and a mixing tip (Pentamix™ 3 Automatic Mixing Unit; 3M ESPE). After mixing, the polysulfide was applied onto the custom tray with a spatula. Meanwhile, the polyvinyl siloxane and the polyether were applied onto the impression tray directly from the mixing tip. Impressions were removed from typodont by lifting the upper base of the tester from the impression tray. To facilitate this procedure, we created an air gap between the impression material and the typodont by inserting a steel spatula between these two surfaces in the external area beyond the perimeter of the buccal fornices. In this way, the procedure was reproducible without altering the area of the impressions relevant to digital analyses.

### 2.3. Digitization of the Conventional Impressions

**Figure 4:**
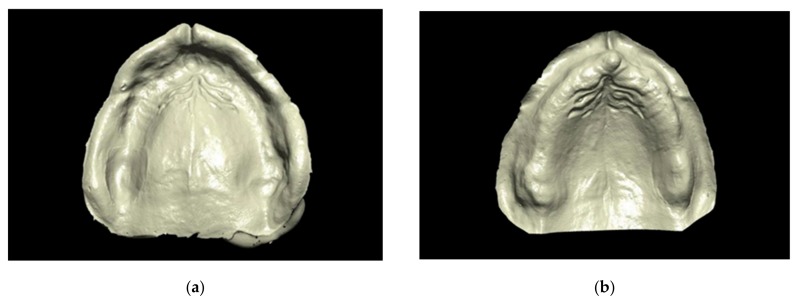
(**a**) Scan of a physical impression obtained from the extraoral laboratory scanner; (**b**) Digital model obtained from the inversion of the scan of a physical impression.

After 30 min, the impressions were removed from the typodont [23], and each of them was eventually scanned using an extraoral laboratory scanner (DScan 3; EGSolutions, Bologna, Italy), employing a structured blue led light. For each experimental group, 10 digital models were obtained using dedicated software (DScan v6.2.2; EGSolutions) after activating the function “Invert Selected Normals” (Figure 4). All the areas needed for the fabrication of a complete maxillary denture was included in the digitization. Three groups of scans were made (n = 10) and respectively named “polysulfide,” “polyvinyl siloxane,” and “polyether.”

### 2.4. Digital Analysis

The .stl files acquired using the extraoral laboratory scanner were imported into a dedicated software (Meshlab v2016.12; ISTI-CNR, Pisa, Italy) using the dRT as a guide for cutting the surplus surfaces of each digital 3D experimental model of the extraoral laboratory scanner. Both the dRT and consequently each digital model was imported into Geomagic Control X (Geomagic Control X v.2018.0.1; 3D SYSTEMS, Morrisville, North Carolina, NC, USA) and then superimposed, choosing the dRT as the software’s “reference data” to determine the accuracy for measuring trueness and precision in μm [24].

An “initial alignment” was done, accompanied by a “best fit alignment,” then the “3D comparison” was enabled. The parameters in the “color bar map” were: max/min range = ±1 mm and specific tolerance = ± 0.1 mm. Eventually, the standard deviation value (SD) was picked from the “tabular view-3D compare”; where this software-based value (SD) represented the mean of positive and negative deviations resulting from each superimposition between the digital surfaces. For this reason, to determine trueness and precision, the mean between SD values was chosen [25].

**Figure 5:**
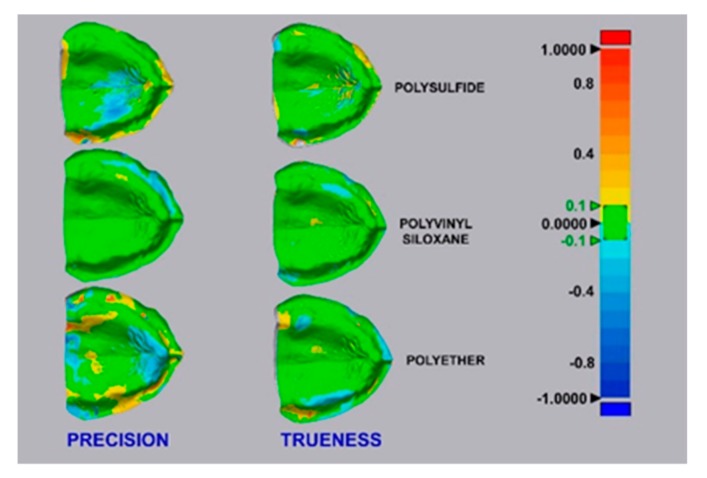
Evaluation of trueness and precision: best superimposition for each group of scans. The green areas indicate a minimum displacement of the digital model compared to the reference data, while red and blue areas indicate an outward and inward displacement between the surfaces, respectively.

A “color map” was generated using this method, for a graphical observation of the displacement between the surfaces of the superimposed digital models. The green areas represent a minimal displacement of ±0.1 mm of the digital model relative to the “reference data”, while the red and blue areas show outward and inward displacements of +1 mm and −1 mm, respectively (Figure 5).

According to ISO-5725, two parameters describe the accuracy of a measurement method: “trueness” and “precision”. Trueness refers to the proximity of agreement between the arithmetic mean of a large number of test results and the reference value. Precision describes the proximity of agreement between intra-group data obtained through repetitive measurements [26,27]. In other terms, trueness determines how a measurement relates to the actual value, while precision represents the consistency of repeated measurements.

For each of the 3 experimental groups, the trueness was calculated as the mean (SD) of each model from dRT. The precision was evaluated as the mean (SD) of each 3D surface model from the model that had obtained the best result of trueness in each of the 3 experimental groups, after superimposing on the dRT. Consequently, all the scans of the same group were superimposed on this selected 3D surface model, and the precision of each experimental group was calculated as the mean (SD) resulting from each of these superimpositions [25].

### 2.5. Statistical Analysis

A dedicated software (IBM SPSS v25; IBM, Armonk, New York, NY, USA) was used to conduct statistical analyses. Both for the analysis of trueness and precision, descriptive statistics (i.e., mean, standard deviation, 95% confidence interval or CI) and specific tests to determine the overall statistical significance of the differences between the groups (p = 0.05). 

In particular, the Shapiro–Wilk test was used to check data normality, the Levene test was conducted to evaluate the homogeneity of variances, and the Kruskal-Wallis test was conducted to analyze differences between groups.

## 3. Results

**Figure 6:**
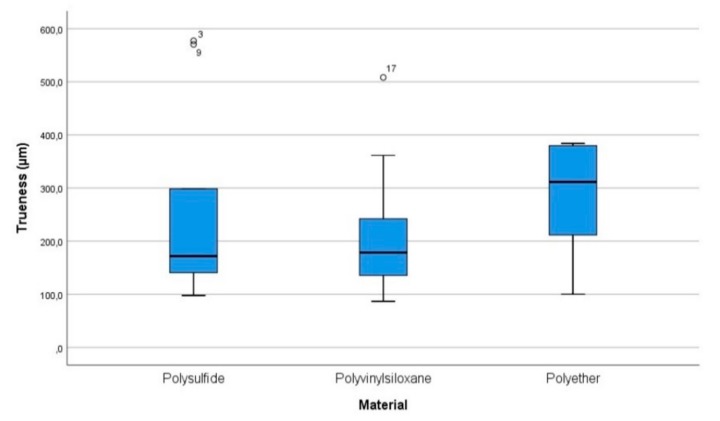
Box plot chart of trueness values. The central rectangle spans the first quartile to the third quartile and whiskers above and below the box show the locations of the minimum and maximum. The segments inside the rectangle shows the median, and unfilled circles represent suspected outliers.

Concerning trueness, the results are summarized in Table 1 and Figure 6. The mean values were not normally distributed for all the groups of scans, as detected by the Shapiro-Wilk test (*p* < 0.05). Levene’s test showed that variances were homogeneous (*p* = 0.272) for the different groups. The Kruskal-Wallis test (*p* = 0.197) showed no statistically significant differences between the mean values of the three experimental groups. Multiple comparisons were not performed because the overall test did not show significant differences across samples.

**Figure 7:**
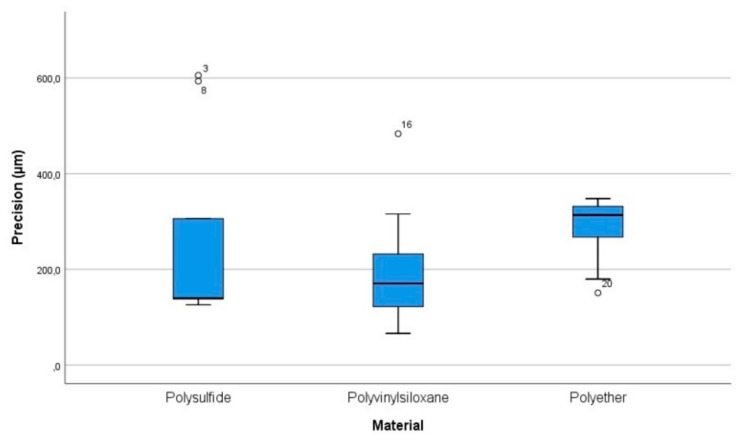
Box plot chart of precision values. The central rectangle spans the first quartile to the third quartile and whiskers above and below the box show the locations of the minimum and maximum. The segments inside the rectangle shows the median, and unfilled circles represent suspected outliers.

Regarding precision, the results are summarized in Table 2 and Figure 7. The mean values were not normally distributed for all the groups of scans, as detected by the Shapiro-Wilk test (*p* < 0.05). Levene’s test showed that there was no homogeneity of variances (*p* = 0.033) for the different groups. A log10 transformation of the data was performed to run a one-way ANOVA. This was because the assumptions of normal distribution and homogeneity of variances were violated. After this transformation, the Shapiro-Wilk test detected again a non-normal distribution (*p* < 0.05) while the Levene’s test reported homogeneity of the variances (*p* = 0.073). The Kruskal-Wallis test (*p* = 0.155) showed no statistically significant differences between the mean values of the three groups. Multiple comparisons were not performed because the overall test did not show significant differences across samples.

The trueness values measured in µm (95% CI) were: polysulfide = 249.9 (121.3–378.5), polyvinyl siloxane = 216.8 (123.1–310.6), polyether = 291.1 (219.9–362.3). The precision values measured in µm (95% CI) were: polysulfide = 261.9 (108.8–415), polyvinyl siloxane = 209.4 (111.9–306.8), polyether = 283 (227.9–338.1).

## 4. Discussion

Removable complete dentures represent the most frequently used typology of prosthetic treatment of total edentulism [4,5,6]. One of the most relevant clinical steps in this kind of rehabilitation is the impression making of the edentulous arches [7].

As previously presented in the Introduction, it is possible to make an impression using different techniques: mucostatic [8], mucocompressive, selective pressure [9], functional [10], and neutral zone impressions [11]. In addition, different impression materials can be used, such as polysulfide, polyvinyl siloxane, irreversible hydrocolloids, zinc-oxide eugenol pastes, and polyethers.

Conventionally, study impressions are made using irreversible hydrocolloids and/or impression compounds, using stock trays [7,28]. Conversely, final impressions are made with zinc-oxide eugenol pastes [12] or with elastomers such as polyethers, polyvinyl siloxanes, or polysulfides, to guarantee a good level of precision [7,13]. Some authors [29] described a third step that aimed to make a circumscribed compression of the tissues to improve prosthetic retention, with the addition of a separate layer of zinc-oxide eugenol for the inner seal [19,29]. 

As reported by Regis et al. [30], a 2-step impression procedure is not mandatory for ensuring clinical success in terms of technical quality, patients’ degree of satisfaction, or improvements in oral health-related quality of life and masticatory function [30].

To date, the use of optical impressions in removable prosthodontics derives from a growing interest in a complete digital workflow for the production of complete dentures [14,15,16,17,18]. Although a few anecdotal studies discussed the use of optical impressions on fully edentulous arches [31,32], according to Mangano et al [33], to date the use of IOSs is contraindicated for the fabrication of complete removable dentures. This is due to the absence of reference points and the impossibility of registering soft-tissue dynamics [33].

Compared to the conventional production process for complete dentures, a digital workflow allows different advantages: better prosthetic fit [34,35,36] and mechanical performance [34]. This is due to a better quality of materials as industrially produced into CAD/CAM blanks, compared to the resin materials pressed into a mold [36] that determine more flaws, porosities, and worse final quality of the base, besides distortions related to materials setting and polymerization shrinkage [34,35,36]. This process saves money and time [34,37], and provides ease of denture duplication due to the accurate reproducibility of the stored digital data [34].

The protocol for a digitally produced complete denture starts with the digitization of an edentulous arch that can be performed using intraoral or laboratory scanners.

With a laboratory scanner, it is possible to obtain digitization by scanning of the physical gypsum model obtained using a conventional impression procedure or scanning the physical impression itself. In this last case, the file is reversed to obtain a positive reproduction of the digital model. Both the procedures performed with a laboratory scanner are to be considered “hybrid” as they require a physical impression, while the use of an IOS does not require any physical gypsum model or physical impression [19].

Further in vitro and in vivo studies will be needed to clarify which impression technique, conventional or digital, is the most accurate, in particular, in the treatment of edentulous patients. Significant differences in the accuracy of digitization were found between different IOSs (mean trueness values ranged from 44.1 to 591.8 μm. Mean precision values ranged from 21.6 to 698.0 μm) [38], between IOSs (video IOS = 197 ± 4 μm; still image IOS = 378 ± 11 μm) and laboratory scanners (170 ± 12μm) [39], and between cone-beam computed tomography (CBCT) scanners (without scanner-spray = 1.2 ± 0.3 μm; with scanner-spray = 1.1 ± 0.2 μm.) and laboratory scanners (without scanner-spray = 4.0 ± 0.3 μm; with scanner-spray = 3.0 ± 0.3 μm) [40].

The present study focused on the first step of the production workflow for complete dentures. It was used as model digitization, obtained by a metrological scanner, as a reference to compare the accuracy of different scans of impressions made using three impression materials: polysulfide, polyether, and polyvinyl siloxane on a reference typodont of a totally edentulous maxilla. 

Until now, only the study by Nedelcu et al. was performed using in vivo a metrological device, but the obtained reference digitization was limited to the buccal surfaces of maxillary anterior teeth and premolars. This was due to the impossibility to use of such a bulky device to perform deeper intraoral scans or to create impressions of edentulous arches [41]. Furthermore, making impression samples directly in the mouth does not guarantee standardized conditions for impression making for the many variables involved in the environmental conditions of the oral cavity. In particular, temperature, humidity, and resilience of soft tissues.

In the present study, the polyurethane resin was chosen for the reference cast because this material has favorable light diffusion and high mechanical resistance [42].

Polysulfide, polyvinyl siloxane, and polyether are supplied as base and catalyst pastes. Particularly, polysulfides are composed of a base paste containing polysulfide polymer (mercaptan with sulfridyl groups -SH), titanium oxide, zinc, sulfate, copper carbonate or silica and dibutyl phthalate. Meanwhile, the catalyst paste is made up of lead dioxide, hydrated copper oxide or organic peroxide, sulfur, oleic acid, or stearic acid. As for polyvinyl siloxanes, the base paste contains polymethylhydrosiloxane and fillers, while the accelerator incorporates divinyl polymethyl siloxane, other siloxane pre-polymers, platinum salt, and retarder. Regarding polyethers, the base paste is composed of polyether polymer with colloidal silica, glycol ether, or phthalate, while the catalyst paste contains alkyl aromatic sulfonate, filler, and plasticizer [43].

The evaluation of trueness and precision obtained using different digitization’s of impressions made in polysulfide, polyether, and polyvinyl siloxane showed that the scans performed directly on the polyvinyl siloxane were more accurate in term of trueness [216.8 (123.1–310.6)] and precision [209.4 (111.9–306.8)] compared to polysulfide [trueness = 249.9 (121.3–378.5); precision = 261.9 (108.8–415)]. Moreover, the polyether showed the worst values of trueness [291.1 (219.9–362.3)] and precision [283 (227.9–338.1)] compared to both the polysulfide and the polyvinyl siloxane. However, it is worth noting that the differences between the means of the experimental groups of scans were not statistically significant. So, it can be inferred that the type of impression material, between the ones tested, did not affect the accuracy of fully edentulous maxilla impressions scanned using a laboratory scanner. This evidence could be explained considering that all three tested materials do not have the physical properties to outperform each other in terms of accuracy on a totally edentulous maxilla.

Polyether is considered favorable for making full-arch impressions in tooth- or implant-supported restorations, and it is a material with a low wetting angle [44]. Compared to polysulfide and polyvinyl siloxane, it shows lower elastic recovery during its removal and high rigidity, together with low flexibility during removal from the oral cavity [7,44,45,46].

Polysulfide exhibits a low wetting angle, good tear strength, and the best flexibility for removal compared to other tested materials. On the contrary, it has low rigidity and elastic recovery [7,45,46].

Finally, polyvinyl siloxane shows the best elastic recovery, but it has a high wetting angle and low tear strength. Moreover, polyvinyl siloxane is more rigid than polysulfide, but it is less than polyether [7,44,45,46].

Such physical properties have to be carefully evaluated during clinical procedures, to select the most appropriate impression material according to the presence of anatomical undercuts and mucosal resilience. 

To date, the clinically accepted accuracy for impressions to fabricate complete dentures has not been established univocally. However, the maximum compressibility of the supporting soft tissues ranges between 0.5–2.0 mm [47]. Therefore, the accuracy required for complete dentures is lower than that required in fixed prosthodontics, in which marginal fit is inside acceptable clinical parameters below 100 μm [48,49,50].

The null hypothesis stating that there was no difference between the accuracy obtained by the various scanning of the 3 experimental materials and that of the reference scan was accepted.

The present study had some limitations, mainly due to its mixed in vitro/in silico nature, because this experimental design did not consider relevant factors related to the oral environment. These factors included humidity, temperature, intraoral anatomic limitations, and the mobility/resilience of soft tissues, affecting the final accuracy of impressions. Further experimental studies involving a larger number of samples should be carried out to shed light on the accuracy of elastomeric impressions on totally edentulous arches.

## 5. Conclusions

Within the limitations of the present in vitro/in silico comparative study, statistically significant differences were not observed between the accuracy of scans performed on the impressions made of polyvinyl siloxane, polysulfide, and polyether on a fully edentulous maxilla. In this research, the different tested materials did not affect the accuracy of a fully edentulous maxilla impression.

Consequently, all the tested materials are clinically suitable to make precise, accurate, and reliable impressions of the fully edentulous maxilla. 

Further, in vitro and in vivo studies, and specifically clinical trials, are needed to validate the results of the present experimentation and identify the most accurate impression material for fully edentulous arches.

## Figures and Tables

**Table 1:** Descriptive statistics for trueness (µm).

Material Scanned	Lower-Upper Bound (95% CI)	Mean	Standard Error
polysulfide	121.3–378.5	249.9	56.8531
polyvinyl siloxane	123.1–310.6	216.8	41.4459
polyether	219.9–362.3	291.1	31.4736

**Table 2:** Descriptive statistics for precision (µm).

Material Scanned	Lower-Upper Bound (95% CI)	Mean	Standard Error
polysulfide	108.8–415	261.9	66.4043
polyvinyl siloxane	111.9–306.8	209.4	42.2547
polyether	227.9–338.1	283	23.8969
